# Brief interventions to address substance use among patients presenting to emergency departments in resource poor settings: a cost-effectiveness analysis

**DOI:** 10.1186/s12962-018-0109-8

**Published:** 2018-06-18

**Authors:** Rebecca Dwommoh, Katherine Sorsdahl, Bronwyn Myers, Kwaku Poku Asante, Tracey Naledi, Dan J. Stein, Susan Cleary

**Affiliations:** 1 Health Economics Unit, School of Public Health and Family Medicine, Faculty of Health Sciences, University of Cape Town, Observatory, Cape Town, 7925 South Africa; 2 Kintampo Health Research Centre, P.O. Box 200, Kintampo, Ghana; 3 Alan J. Flisher Centre for Public Mental Health, Department of Psychiatry & Mental Health, University of Cape Town, Cape Town, 7925 South Africa; 4 Department of Psychiatry & Mental Health, Groote Schuur Hospital, University of Cape Town, Cape Town, 7925 South Africa; 5 Alcohol, Tobacco, and Other Drug Research Unit, South African Medical Research Council, Tygerberg, 7505 South Africa; 6 Western Cape Department of Health, 8 Riebeeck Street, Cape Town, 8001 South Africa; 7 South African MRC Unit on Risk & Resilience in Mental Disorders, Department of Psychiatry and Mental Health, University of Cape Town, Cape Town, 7925 South Africa

**Keywords:** Substance use, Emergency departments, Brief interventions, Problem solving therapy, Cost-effectiveness analysis

## Abstract

**Background:**

There are limited data describing the cost-effectiveness of brief interventions for substance use in resource-poor settings. Using a patient and provider perspective, this study investigates the cost-effectiveness of a brief motivational interviewing (MI) intervention versus a combined intervention of MI and problem solving therapy (MI-PST) for reducing substance use among patients presenting to emergency departments, in comparison to a control group.

**Methods:**

Effectiveness data were extracted from Project STRIVE (Substance use and Trauma InterVention) conducted in South Africa. Patients were randomised to either receive 1 session of MI (n = 113) or MI in addition to four sessions of PST (n = 109) or no intervention [control (n = 110)]. Costs included the direct health care costs associated with the interventions. Patient costs included out of pocket payments incurred accessing the MI-PST intervention. Outcome measures were patients’ scores on the Alcohol, Smoking and Substance Use Involvement Screening Test (ASSIST) and the Centre for Epidemiological Studies Depression Scale (CES-D).

**Results:**

Cost per patient was low in all three groups; US$16, US$33 and US$11, and for MI, MI-PST and control respectively. Outcomes were 0.92 (MI), 1.06 (MI-PST) and 0.88 (control) for ASSIST scores; and 0.74 (MI), 1.27 (MI-PST) and 0.53 (control) for CES-D scores. In comparison to the control group, the MI intervention costs an additional US$119 per unit reduction in ASSIST score, (US$20 for CES-D); MI-PST in comparison to MI costs US$131 or US$33 per unit reduction in ASSIST or CES-D scores respectively. The sensitivity analyses showed that increasing the number of patients who screened positive and thus received the intervention could improve the effectiveness and cost-effectiveness of the interventions.

**Conclusion:**

MI or MI-PST interventions delivered by lay counsellors have the potential to be cost-effective strategies for the reduction of substance use disorder and depressive symptoms among patients presenting at emergency departments in resource poor settings. Given the high economic, social and health care cost of substance use disorders in South Africa, these results suggest that these interventions should be carefully considered for future implementation.

*Trial registration* This study is part of a trial registered with the Pan African Clinical Trial Registry (PACTR201308000591418)

## Background

Substance use disorders represent a major public health problem. Globally, alcohol and illicit drug use are reported to be the cause of death for over 350,000 and 40,000 people respectively per year [1]. Alcohol and other drugs use disorders account for 8 and 10% of the Disability Adjusted Life Years (DALYs) attributable to mental and behavioral disorders respectively [2]. Results from the South African Stress and Health Study (SASH), the first nationally-representative study of psychiatric morbidity in South Africa, indicate a high lifetime prevalence (13.3%) and early onset (21 years) of substance use disorders [3]. Despite this high prevalence, only about 10% ever receive treatment [4, 5].

Service delivery for the treatment of substance use disorders in low and middle income countries, including South Africa, has been criticized. When available, treatment is usually oriented to tertiary treatment of severe substance use disorders with an emphasis on long-term residential treatment [6, 7]. The treatment gap in South Africa could be narrowed by broadening the base of treatment and by targeted screening and brief psychotherapeutic interventions for those most at risk. Data from developed countries have revealed that brief interventions for alcohol use disorders are effective, feasible, and cost-effective in emergency departments [8, 9] although there is presently far less data available for other types of substance use disorders. For example, 9 out of 14 studies in a review concluded that brief interventions were effective in reducing alcohol consumption, hazardous use of alcohol, and alcohol-related injuries in comparison with usual emergency department care [10]. Additionally, in a cost–benefit analysis of brief interventions in emergency department patients, the net cost saving of the intervention per patient screened was US$89, or US$330 per patient offered an intervention [9]. Whilst there are a number of studies available from developed settings, there has been little work evaluating these interventions in low and middle income countries (LMICs) despite the high prevalence of substance use disorders in these countries. Such evaluations can inform policy makers on the need to invest in these interventions given their benefits.

To address this gap, the cost-effectiveness of two alternative brief interventions administered by lay counsellors to patients presenting at emergency departments in Cape Town in the Western Cape Province of South Africa, in comparison to a control group, was evaluated. The Western Cape Province has the highest lifetime prevalence of substance use disorder in South Africa with a prevalence of 20.6% compared to 13.3% nationally [11].

## Methods

The aim of this study was to assess the cost-effectiveness of two brief interventions—motivational interviewing (MI) and a combined intervention of MI and problem solving therapy (MI-PST)—administered by lay counsellors to reduce substance use among patients presenting to emergency departments in resource poor settings, in comparison to a control group.

### Study design

This cost-effectiveness analysis was conducted as part of Project STRIVE (Substance use and Trauma InterVention), a randomized control trial comparing two brief interventions to a control group. The MI-PST intervention is a combination of problem-solving therapy with motivational interviewing while the MI intervention offered just motivational interviewing to patients attending emergency departments in the Western Cape province of South Africa. The interventions were offered by lay counsellors and patients were followed up at 3 months post-baseline to assess the effectiveness of the interventions [12]. Cost-effectiveness was assessed from the societal perspective. Economic costs were calculated for the provider perspective in each arm, and a subset of patient costs were assessed in the MI-PST arm. Ingredients and step down methods were used to calculate the economic cost of the interventions and cost-effectiveness was assessed by computing incremental cost-effectiveness ratios (ICERs). The ICER is the ratio of additional costs to additional effects—comparing each costlier intervention to the one directly preceding it [13]. Given that all costs and outcomes occurred within a 1-year period, neither costs nor outcomes were discounted. A range of one-way sensitivity analyses and scenario analyses were conducted to examine the robustness of the study findings. Analysis was undertaken using Microsoft Excel and TreeAge Pro 2013, R1.0. All costs were calculated in 2012/13 prices and were converted to United States Dollars (US$) using an exchange rate from the same period (US$1 = ZAR 10.3952) [14].

### Study setting

Participants were recruited within the emergency departments of Khayelitsha Site B Community Health Centre, Khayelitsha District Hospital and Elsie’s River Community Clinic, all located within the Western Cape Province. The unemployment rate in the Province is about 21% [15]. The Province also has a high rate of substance use, crime and violence [16].

### Screening, recruitment and assessment

Across the three emergency departments, lay counsellors screened 2736 consenting patients using the Alcohol, Smoking, and Substance Involvement Screening Test (ASSIST) while they waited to see a healthcare provider. Based on ASSIST scores, patients were classified as low risk (with a score of 0–10 for alcohol and < 4 for other drug use), moderate risk (with a score of 11–26 for alcohol and 4–26 for other drug use) or high risk (with scores above 26 for alcohol and other drugs use) [12]. Of the total screened, 531 (19%) patients screened positive for moderate to high risk of substance use, of whom 332 (63%) agreed to be included in the intervention program. Patients were randomly assigned to a single session intervention based on motivational interviewing (MI; n = 113), a blended motivational interviewing and problem solving intervention (MI-PST) comprising five counselling sessions (n = 109) or a control group (n = 110). Patients at high risk were referred for specialised treatment whilst those at low risk were excluded from the study. Patients excluded from the study were not significantly different from the eligible patients. Those who refused to participate in the study had significantly lower ASSIST scores (M = 15.68, SD = 7.60) compared to those who were willing to participate in the study (M = 17.45, SD = 9.08) [12].

In addition to the patient’s level of substance use involvement, their depressive symptoms were assessed at baseline with the Centre for Epidemiological Studies Depression Scale (CES-D) [15]. This scale measures common symptoms of depression based on 20 self-rated items. Each item is graded on a four-point Likert scale, ranging from 0 (representing presence of no depressive symptom) to 3 (indicating the presence of depressive symptoms most of the time). Composite scale scores range from 0 to 60, with a score of 16 or higher representing clinically meaningful depression [12, 17].

These assessments were re-administered at 3 months to determine the effectiveness of the interventions. This was done for 182 participants [control group = 66; MI group = 70; MI-PST group = 46] who were available for the 3 months follow-up assessment. These formed 54% of the entire study sample. More details on the screening, recruitment and assessment of the outcomes are provided in the published trial paper [12].

### Counsellors

The counsellors used for the study were recruited from the study areas. They had tertiary level education or equivalent knowledge, and were trained specifically to screen and offer the interventions. In addition to being trained, they participated in biweekly supervision and debriefing sessions throughout the study period [12, 18].

### Description of interventions

#### MI intervention

Patients randomised to the MI group received one session of MI directly after screening and baseline assessment. This session lasted about 30 min in total and was based on the ASSIST linked brief intervention [19]. During this session, lay counsellors provided feedback on the patient’s level of risk based on their ASSIST score, discussed the patient’s substance use patterns and the importance of moderating behaviour in order to minimise health and other risks. Through a motivational interviewing approach, they enhanced the patient’s motivation and willingness to change. Patients received MI-specific substance use risk cards which summarized their risks, in addition to the substance misuse fact sheet and the contact details of local support centres [12].

#### MI with PST intervention

Patients assigned to this intervention group received the same MI intervention as outlined above in addition to four sessions of PST provided on a weekly basis. These PST sessions were designed to help patients cope better with psychological stress and the other stresses of daily life [20, 21]. The first PST session lasted about 60 min while the other three lasted 40 min each. The content of these sessions has been described in detail elsewhere [12, 18].

#### Control

While no additional psychotherapeutic support was offered to patients randomized to this group, in an improvement over usual care, patients were provided with a leaflet providing information on the effects of substance use and the contact details of local support centres [12].

#### Outcome measures

Outcome measures for the study were patients’ scores on the ASSIST and the CES-D. As explained above, these measure patient’s risk of substance use and depressive symptoms respectively. Mean scores for patients in each of the intervention and control groups were calculated at baseline and at the 3 months follow-up. The differences in the means at baseline and the 3 months follow-up for each of the groups were calculated and compared to establish the effectiveness of the interventions. For patients who used more than one substance, reduction in ASSIST scores for the primary substance was used in the analysis.

#### Provider costs

The scope of provider costs included all cost items needed to deliver the interventions including staffing (counsellors and psychologists), supplies (screener tools, flyers, manuals, fact sheets, pens, tapes and tape recorders for fidelity checks) and capital (counselling room, furniture and training). Research costs (e.g. administering baseline and follow-up assessments) were excluded. The costs were categorized as variable or fixed, and are summarized in Table 1. Table 1 also summarizes the unit cost of each resource and quantity consumed.

**Table 1:** Provider costs

Cost item	Details	Cost (US$)
Variable costs		
Staffing		
Supervisor (direct counsellor support)	Bi-weekly supervision over 9 months	562.76
Supervisor (fidelity checks)	5 h per week over 9 months	25,973.53
Manuals, tools, flyers		
Screening tool	1 per person screened	0.10
Substance misuse flyer	1 per person accepting intervention	0.08
MI manual	1 per person receiving MI	0.34
PST manual	1 per person receiving MI-PST	2.00
Fixed costs		
Staffing		
Counsellors	5 staff employed over 9 months	25,973.53
Supplies		
Pens	10 per counsellor	0.46
Clipboards	1 per counsellor	1.54
Tape recorders (fidelity checks)	1 per counsellor	115.44
Tapes (fidelity checks)	Amount used over 9 months	346.31
Capital (annuitized using 3% discount rate)		
Counselling room	1 room in each emergency department	220.26
Furniture	Desk and 2 chairs in each room	285.08
Counsellor training	45 h’ training including screening, MI and PST	425.11

Variable costs are those that vary with scale. Under staffing, these include the clinical psychologist that was contracted to undertake bi-weekly supervision and fidelity checks, and whose payment was related directly to hours worked. Variable costs also include the manuals, tools and flyers that were distributed to each client as appropriate to the intervention arm. Fixed costs, on the other hand, are assumed to stay constant over the short term. For the purposes of this costing, these include certain supplies (pens, clipboards, tapes and tape recorders for fidelity checking), capital costs (counselling room space, furniture, and training) and counsellor salary costs.

Capital costs included the cost of counsellors’ training, room space and furniture used within the facilities. The interventions were administered in private rooms in the emergency departments. Cost of room space was estimated based on the price index for new buildings in South Africa [22]. Cost of furniture was estimated based on market prices. It was assumed that the useful life for furniture and buildings were 5 and 20 years respectively. Total cost of counsellor training was estimated as the cost of the training materials, room space, and the salary of the trainer. Retraining for lay counsellors was assumed to be within the next 3 years. Capital costs were annuitised using a 3% discount rate [13], chosen to facilitate comparability with other cost-effectiveness analyses. The nature of this intervention (offered at emergency departments that are open 24 h/day, 7 days/week) meant that 5 counsellors were employed in total across the 3 facilities, with implications for economies or diseconomies of scale within the costing calculations. Counsellor salary costs were therefore assessed as fixed costs, and variation was explored within sensitivity/scenario analysis.

#### Allocation of provider costs to interventions

The costs of manuals, screeners and flyers were allocated directly to patients based on their utilization within each intervention arm. All other costs were allocated to screening, MI and MI-PST based on the amount of time that counsellors spent on these different components. In essence, the patient contact time (in minutes) for each session (screening, MI and MI-PST) was calculated and multiplied by the number of each type of session. The proportion of total time spent screening, offering the MI intervention, or the MI-PST intervention was then used to allocate costs to intervention components. These calculations are presented in Table 2.

**Table 2:** Estimated counsellor time spent on different intervention components

Intervention elements	Minutes per patient	Number of sessions	Total minutes	Share of time (%)
Screening	10	2736	27,360	57
MI group				
MI session	30	113	3390	7
MI-PST group				
MI session	30	109	3270	36
First PST session	60	87	5220	
Second PST session	40	77	3080	
Third PST session	40	69	2760	
Fourth PST session	40	65	2600	
Totals		3256	47,680	100

#### Patient costs

Costs to patients for participating in the intervention included the transportation costs incurred when travelling to the facilities to attend their PST sessions, productivity costs in terms of salary loss, cost of care takers and other non-health care costs to patients as a result of their participation in the intervention. These costs were only incurred by patients in the MI-PST group, given that patients in the other arms were counselled while present at the emergency department for treatment for their injuries.

#### Estimation of intervention costs and cost-effectiveness

**Fig. 1:**
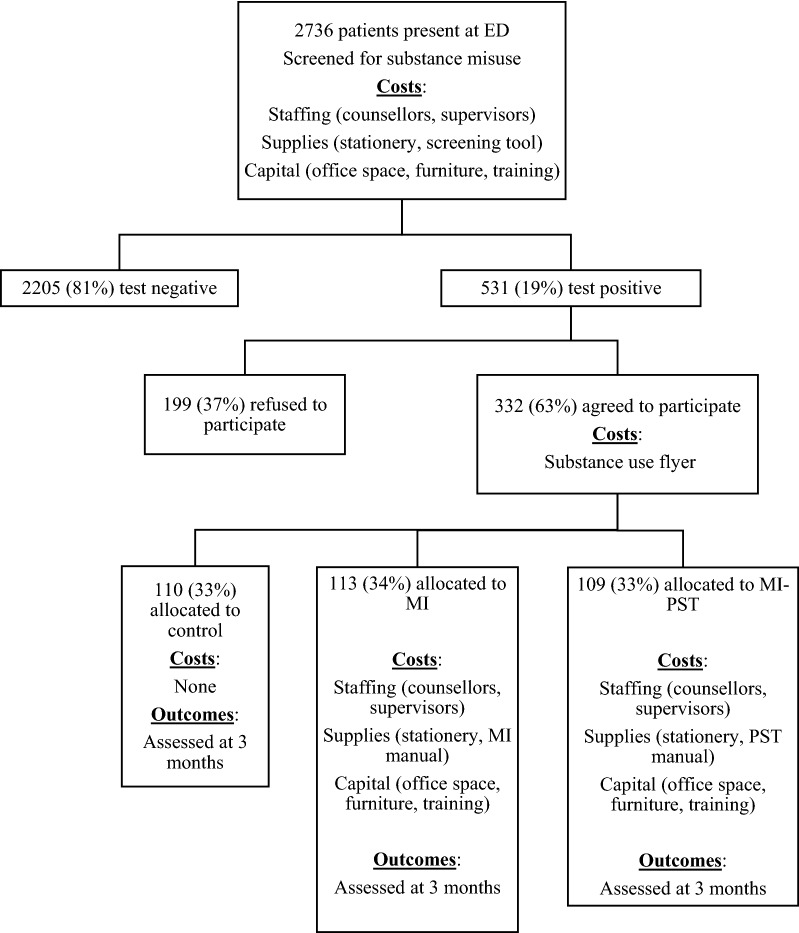
Intervention flow chart

Once costs had been allocated to intervention components, they were allocated to interventions to estimate the total costs of screening and each of the interventions. For example, screening costs were incurred for all 2736 participants screened, and were allocated equally to each intervention group, while MI costs were allocated to the MI group, MI-PST costs were allocated to the MI-PST group etc. The allocation of costs is visually depicted in Fig. 1, which replicates the structure of the decision tree employed for this analysis (created in TreeAge Pro 2013, R1.0). The decision tree is an appropriate modelling approach for interventions unfolding over short time periods [23]. In the model, patients were allocated to the intervention groups (MI only, MI with PST and status-quo of no intervention (control)) and followed through a series of events to establish the costs and outcomes of the interventions. In assessing cost-effectiveness, the model took into account patients who were lost to follow-up, excluded or refused the intervention. Costs and outcomes for patients in the MI-PST intervention group for instance were analysed based on whether they dropped out of the intervention or not and the number of PST sessions they attended. The model estimated the incremental cost effectiveness ratios (ICERs) of the interventions. The ICER is the ratio of additional costs to additional effects—comparing each more costly intervention to the one directly preceding it [13]. It compares the difference in costs and effectiveness of the competing interventions as illustrated in Eqs. (1) and (2) below:

1 $${\text{ICER}}_{\text{YX}} = \Delta {\text{C/}}\Delta {\text{E}}$$

2 $$\Delta {\text{C}} = {\text{C}}_{\text{Y}} - {\text{C}}_{\text{X}} ;\quad \Delta {\text{E}} ={\text{E}}_{\text{Y}} - {\text{E}}_{\text{X}}$$

where C_Y_ is the cost of intervention Y (the more costly intervention) and E_Y_ is its effectiveness; C_X_ and E_X_ are the costs and effectiveness of intervention X (the less costly intervention).

#### Sensitivity analysis

One-way sensitivity analysis was conducted to test the robustness of the study findings [24] by varying key cost and outcome drivers. Testing the generalizability of the findings to other (non-study) settings was of particular interest. The proportion of patients screening positive for substance misuse, the proportion of patients accepting participation in the interventions and the extent of excess capacity within the fixed costs (counselling time, supplies and capital) were varied. The impact of these changes on incremental costs, the incremental ASSIST score and incremental cost-effectiveness ratios were captured.

## Results

### Baseline characteristics

Men constituted 65% (n = 218) of the study participants. Their ages ranged between 18 and 75 years with the average age being 28 years. 72.2% of the participants attended the emergency department for treatment of injuries while the remainder sought care for ill health. Alcohol was the most common substance used (n = 286, 85%), followed by cannabis (n = 24, 7%) and methamphetamine (n = 20, 6%) [10].

### Effectiveness

All three groups recorded some reduction (i.e. improvement) in their outcome measures. Patients in the control group recorded mean reductions of 7.39 and 4.43 points for ASSIST and CES-D scores respectively, whilst those who received the MI intervention recorded a mean reduction of 7.68 for the ASSIST and 6.16 for the CES-D. Participants who received the MI-PST intervention recorded significantly better outcomes (p < 0.001) than those in the MI or control group, with a mean reduction of 8.82 and 10.64 for ASSIST and CES-D scores respectively. More details are available in the published trial paper [12]. The mean scores at baseline, follow-up and mean reductions are presented in Table 3.

**Table 3:** Effectiveness of interventions (showing mean scores at baseline, 3 month follow-up and mean reduction)

	Control mean (SD)	MI mean (SD)	MI-PST mean (SD)
Substance use: ASSIST			
Baseline	19.30 (5.78)	19.96 (6.49)	18.71 (6.32)
3 month follow-up	11.91 (6.94)	12.28 (6.81)	9.89 (6.64)
^a^Mean reduction	7.39	7.68	8.82
Depression: CES-D			
Baseline	24.56 (6.02)	23.93 (5.43)	27.28 (8.22)
3 month follow-up	20.13 (7.09)	17.77 (8.12)	16.64 (8.17)
^a^Mean reduction	4.43	6.16	10.64

*SD* standard deviation

^a^Mean reduction is the difference between scores at baseline and follow-up

Overall, 182 (54%) participants completed the 3 month follow-up: 70 (62%) in the MI, 46 (42%) in the MI-PST and 66 (60%) in the control arms. Loss to follow-up was highest in the MI-PST group. Of those who did not complete the MI-PST, 3 discontinued because they were admitted to tertiary care and were not available for the follow-up. The distinguishing variables between those who completed the 3 month follow-up and those who did not, were treatment condition (*χ*^*2*^=10.97, df = 2, p < 0.001) and race (*χ*^*2*^=9.04, df = 2, p = 0.011). Patients of the Coloured race were more likely than those of the Black African race to complete the follow-up assessment. Similarly, those in the MI arm were more likely than those in the other arms to complete the follow-up assessment [12].

### Provider costs

The provider cost per patient includes screening, MI, and MI-PST costs as appropriate for patients accepting to participate. It also includes the costs spent on screening those who were negative; these costs have been allocated equally to the three groups. If all positively screened patients accepted to participate and completed all intervention activities, the average costs per patient would be US$95 in the control group (comprising screening costs), US$129 for the MI intervention (includes screening and MI intervention costs) and US$279 for the MI-PST intervention (includes screening and MI-PST costs), as summarized in Table 4.

**Table 4:** Average cost per patient screened and treated

Cost measure	Average cost per patient		
	Control	MI	MI-PST
Provider costs			
Cost of screening	US$95	US$95	US$95
Cost of the intervention		US$34	US$184
Sub Total	US$95	US$129	US$279
Patient costs			
Cost of transportation	N/A	N/A	US$3 (cost per visit = US$0.96)
Total cost per patient	US$95	US$129	US$282

These costs would be incurred if everyone accepted participation and completed all intervention components

N/A = patient costs were only incurred for those returning to the facility for PST sessions

### Patient costs

Patients were interviewed about the direct and indirect non health care costs that they incurred in accessing PST sessions. As mentioned earlier, these costs exclude any costs that may have been incurred in accessing the emergency department at baseline as these would be classified as injury associated costs instead of intervention costs; once at the emergency department, it was assumed that any increase in waiting time needed to access screening and MI would be negligible. However, for patients in the MI-PST group, intervention costs may be incurred as patients needed to travel back to the emergency department on a weekly basis to attend their counselling sessions. These costs were however reported to be very small. Of the 48 patients interviewed, 85% walked to the facility, incurring zero transport costs; across the sample, the mean transport payment was US$0.96 per visit. 8% reported taking time off work to attend the session, but this lead to a loss in income for only 2%.

### Cost-effectiveness

Table 5 summarizes costs, effects and ICERs for baseline parameter estimates. Cost per patient is low in all three intervention groups (US$11, US$16 and US$33 for control, MI and MI-PST respectively). These costs are lower than the average costs presented in Table 4 because they are influenced by the percentage (63%) accepting to participate in the interventions. Outcomes are 0.88 (control), 0.92 (MI) and 1.06 (MI-PST) for ASSIST scores; and 0.53 (control), 0.74 (MI), and 1.27 (MI-PST) for CES-D scores. In comparison to the control group, the MI intervention costs an additional US$119 per unit reduction in ASSIST score, and US$20 per unit reduction in CES-D score; MI-PST in comparison to MI costs US$131 or US$33 per unit reduction in ASSIST or CES-D scores respectively.

**Table 5:** Baseline cost-effectiveness results

Interventions	Cost	Incremental cost [ΔC]	Outcome		Incremental outcome [ΔE]		Incremental cost effectiveness ratios (ICERs) [ΔC/ΔE]	
			AS	DP	AS	DP	AS	DP
Control	11.44	–	0.88	0.53	–	–	–	–
MI	15.57	4.13	0.92	0.74	0.03	0.21	118.96	19.94
MI-PST	33.48	17.91	1.06	1.27	0.14	0.54	131.25	33.40

*AS* ASSIST score, *DP* CES-D, *ΔC* change in cost (compared to preceding intervention), *ΔE* change in effectiveness (compared to preceding intervention), *MI* motivational interviewing, *PST* problem solving therapy

As explained, the proportion testing positive for substance misuse and accepting participation significantly influences the magnitude of these costs; this potentially happens in two ways. Firstly, higher positivity and acceptance rates mean that screening costs will generate a greater ‘yield’, reducing the cost per patient screened positive; secondly, with increased scale, costs such as the counselling costs for MI and MI-PST would theoretically increase. However, this would only be the case in the instance of zero excess counselling capacity. If excess capacity exists, increased patient numbers have the impact of *reducing* MI/MI-PST costs on a per patient basis in that these staff become busier, which is the more relevant scenario, given the high levels of excess capacity found in the fixed costs in this study.

As shown in Table 2, five counsellors conducted a total of 3256 patient sessions (screening, MI and PST) spending an estimated 47,680 min (795 h) in total. In contrast, if we assume that it is reasonable for each counsellor to spend 6 h counselling patients each working day, the total amount of counsellor time available (assuming routine leave entitlements) would amount to 4905 h, suggesting that a total of 16% of their available capacity was utilized during the study period. Put differently, counsellors had the potential to accommodate an 84% increase in patient load. This finding is further explored in sensitivity analysis.

### Sensitivity analysis

**Fig. 2:**
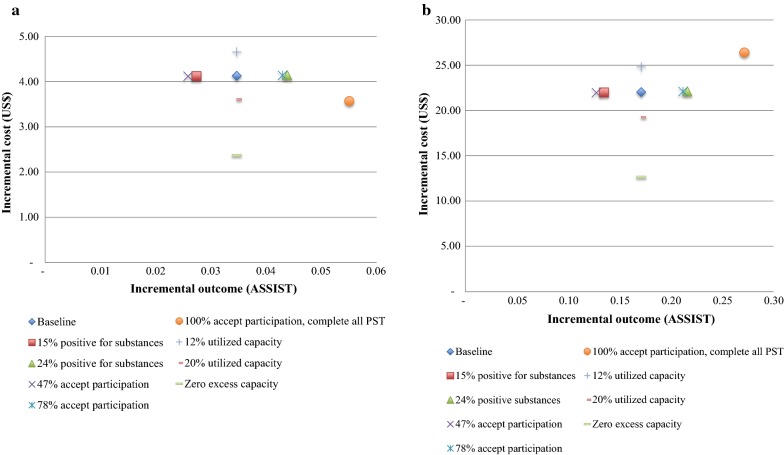
**a** MI sensitivity analyses (compared to control), **b** MI-PST sensitivity analyses (compared to control)

Figure 2a, b summarize the results of sensitivity analyses for changes in incremental costs, incremental outcomes (ASSIST) and the ICER. Analyses include (1) the proportion screening positive for substance misuse (varying the baseline rate of 19 by 25% in each direction); (2) the proportion accepting participation in the interventions (again varying the baseline rate of 63 by 25% in each direction, and exploring an extreme scenario where 100% accept participation and where participants in the MI-PST group complete all of their PST sessions); and (3) the extent of excess capacity (varying the baseline rate (84%) by 25% in each direction and exploring an extreme scenario of zero excess capacity in fixed costs). In each analysis, findings are represented relative to the control group (i.e. MI versus control and MI-PST versus control).

In both interventions, assuming zero excess capacity in fixed costs generates the highest cost savings. In this scenario, it is assumed that counsellors spend at least 6 h/day seeing clients, which simultaneously increases the efficiency of the utilization of capital inputs (counselling room, furniture, training) and supplies (tape recorders etc.). Similarly, if 100% of clients screening positive for substance misuse accept the intervention and if all PST sessions are completed, costs are reduced (owing to reduced excess capacity), outcomes increase and cost-effectiveness improves. If fewer clients test positive for substance misuse, and if fewer accept participation, this would reduce costs and reduce outcomes, with the net impact of decreasing cost-effectiveness.

## Discussion

This study examined the cost-effectiveness of two brief interventions delivered in emergency departments by counsellors, in comparison to a control group. The results show that both MI and MI-PST require additional resources and both have the potential to improve outcomes in comparison to the control group. The cost per patient is however low, and should be contrasted with the cost of treating the injuries that could be averted through effective substance use interventions in this setting. For example, at US$36, the cost per emergency department visit is higher than the cost per patient for MI (US$16) or MI-PST (US$33). Any further judgement regarding which (if any) intervention represents value for money can only be made through conducting a cost-utility analysis and comparing the ICERs to an external threshold [25].

The sensitivity analyses indicate that gains in cost-effectiveness can be made through targeting the interventions to emergency departments where high proportions of patients present with substance use disorders, by increasing the extent to which clients accept participation in the interventions, and by reducing counselling excess capacity. Given the study design, counsellors were employed solely to deliver these interventions; in routine settings it would be beneficial to explore options where counsellors would offer substance use interventions in addition to other counselling services so that their capacity could be utilized more efficiently.

The study highlights that, at relatively minimal costs, it is possible to employ and train people without any prior medical or mental health skills to screen and effectively deliver the brief interventions in emergency departments. Expert consensus indicates that the cost of employing a trained clinical psychologist for these activities could be a lot more than that for the counsellors used for this study. This supports evidence that task-shifting and sharing is a cost effective and efficient way of expanding access to mental health care including substance use disorders [26–28].

The results of this study support findings from other studies that brief interventions for substance use in emergency departments can be relatively affordable and effective [29–31]. The inclusion of a combined MI with PST intervention in the analysis differentiates the current study from other published studies. Even though the analysis of brief MI for substance use is common at least in developed countries, the same cannot be said about PST, let alone a combination of the two interventions in a resource poor setting. This study has therefore shown that administering a combined intervention of MI with PST can be a better option than just the MI only intervention, especially if the aim is to reduce patient’s substance use and depressive symptoms.

Despite the promising results of the cost-effectiveness analysis, the ICERs presented cannot form the only basis upon which implementation decisions are made. In effect, as suggested by Birch and Gafni [32], a consideration of the total cost of the interventions, the number of people at risk of substance use disorders, those in need of the interventions, the general health needs of the population and the available health budget must be considered in decision making.

Given the level of unemployment in the study setting, it is unsurprising that very few participants reported productivity losses. Of those who were employed, only four took time off work to attend their sessions and only one reported losing some salary. In South Africa, public sector services such as those considered in this study, are free at the point of use. On average, patients reported spending US$0.96 per visit in travel costs, which is small in comparison to the provider costs that were incurred. Whilst costs to patients are lower in our study setting, these could be higher in other settings where patients could incur higher costs of productivity losses, transportation and payment for services. These could impede patients’ utilization of these services. In such settings there would be the need for support systems such as government subsidies or insurance coverage to encourage utilization.

There was a high rate of loss to follow-up in the MI-PST intervention group and this is expected in routine settings. The overall results were sensitive to variations in loss to follow up during the MI-PST intervention. In a sensitivity analysis, the proportion of patients that agreed to participate in either intervention was varied, and it was assumed that all patients completed their PST sessions. This resulted in improved effectiveness and cost-effectiveness.

A limitation of this study, shared by many other cost-effectiveness studies of brief interventions for substance use, is that the outcome measures used cannot be compared to that of other studies [33]. This is mostly because there is no agreed upon outcome measure for use in cost-effectiveness analysis of brief interventions for substance use [34]. In spite of this, the study provides useful information on the cost-effectiveness of these brief interventions to guide decision making in a resource poor setting.

Another limitation is the short timeframe of the study. Three months may not be long enough to establish the full economic costs and effects of the interventions. Despite the encouraging results presented in this study, it would have been beneficial to do a longer term study that assesses the long term outcomes and economic costs of the interventions. These interventions have been shown to be effective in reducing substance use [12] and thus have the potential to impact on the utilisation of emergency department and other health and social care services. Reduced substance use could also be valuable for the wellbeing of communities through reduced violence and crimes. Future studies estimating Quality Adjusted Life Years (QALYs) as well as the economic costs of these interventions could enable comparisons across different interventions [35] and address questions of value for money [25].

## Conclusion

This study has illustrated that the use of lay counsellors for screening and the administration of brief interventions for reducing substance use in emergency departments can be effective at a relatively low cost. In resource poor settings where substance use is high and resources are limited both in terms of finance and well qualified human resources, introduction of interventions like this can be useful in minimising substance use and related problems. Additionally, given the high economic, social and health care cost of substance use disorders in South Africa [36], these results suggest that this intervention should be carefully considered for future implementation. These are the first data from an economic evaluation of a combined intervention of MI with PST in a resource poor setting and provide the basis for future work that could explore the potential long term effects of the interventions in terms of averting the health, economic and social costs of substance use disorders.

## Abbreviations

ASSIST: Alcohol, Smoking, and Substance Involvement Screening Test
CES-D: Center for Epidemiological Studies Depression Scale
ICERs: incremental cost-effectiveness ratios
LMICs: low and middle income countries
MI: motivational interviewing
PST: problem solving therapy
QALYs: Quality Adjusted Life Years
SASH: South African Stress and Health Study
STRIVE: Substance use and Trauma InterVention

## References

[1] World Health Organization. Atlas on substance Use (2010): Resources for the prevention and treatment of substance use disorders. World Heal Organ. 2010:156.

[2] WHO Forum on alcohol, drugs and addictive behaviours Enhancing public health actions through partnerships and collaboration: Alcohol and drug use disorders: Global Health Estimates [http://www.portal.pmnch.org/substance_abuse/activities/fadab/msb_adab_2017_GHE_23June2017.pdf] Accessed 8 May 2018.

[3] Stein DJ, Seedat S, Herman A, Moomal H, Heeringa SG, Kessler RC (2008). Lifetime prevalence of psychiatric disorders in South Africa. Br J Psychiatry.

[4] Meade CS, Towe SL, Watt MH, Lion RR, Myers B, Skinner D (2015). Addiction and treatment experiences among active methamphetamine users recruited from a township community in Cape Town, South Africa: a mixed-methods study. Drug Alcohol Depend.

[5] Myers B, Kline TL, Doherty IA, Carney T, Wechsberg WM (2014). Perceived need for substance use treatment among young women from disadvantaged communities in Cape Town, South Africa. BMC Psychiatry..

[6] Perngparn U, Assanangkornchai S, Pilley C, Aramrattana A (2008). Drug and alcohol services in middle-income countries. Curr Opin Psychiatry..

[7] Myers B, Sorsdahl K (2014). Addressing substance use within primary health care settings in South Africa: opportunities and challenges. Addicta Turk J Addict..

[8] Schermer CR (2005). Feasibility of alcohol screening and brief intervention. J Trauma Acute Care Surg..

[9] Gentilello LM, Ebel BE, Wickizer TM, Salkever DS, Rivara FP (2005). Alcohol interventions for trauma patients treated in emergency departments and hospitals: a cost benefit analysis. Ann Surg.

[10] Nilsen P, Baird J, Mello MJ, Nirenberg T, Woolard R, Bendtsen P (2008). A systematic review of emergency care brief alcohol interventions for injury patients. J Subst Abuse Treat.

[11] Herman AA, Stein DJ, Seedat S, Heeringa SG, Moomal H, Williams DR (2009). The South African Stress and Health (SASH) study: 12-month and lifetime prevalence of common mental disorders. South Afr Med J (SAMJ).

[12] Sorsdahl K, Stein DJ, Corrigall J, Cuijpers P, Smits N, Naledi T (2015). The efficacy of a blended motivational interviewing and problem solving therapy intervention to reduce substance use among patients presenting for emergency services in South Africa: a randomized controlled trial. Subst Abuse Treat Prev Policy..

[13] Drummond MF, Sculpher MJ, Torrance GW, O’Brien BJ, Stoddart GL (2005). Methods for the economic evaluation of health care programmes.

[14] Exchange rate: Rands to Dollars. https://www.Oanda.com. Accessed July 2016.

[15] Lehohla P. Census 2011 Municipal report Western Cape. 2012. http://www.statssa.gov.za/census/census_2011/census_products/WC_Municipal_Report.pdf. Accessed 18 Jan 2014.

[16] Western Cape Crime Overview 2014/15 Analysis of Crime Statistics as released by the South African Police Service on 29th of September 2015. 2015.

[17] Radloff LS (1977). The CES-D scale: a self-report depression scale for research in the general population. Appl Psychol Meas.

[18] Myers B, Stein DJ, Mtukushe B, Sorsdahl K (2012). Feasibility and acceptability of screening and brief interventions to address alcohol and other drug use among patients presenting for emergency services in Cape Town, South Africa. Adv Prev Med..

[19] Organization WH (2010). The alcohol, smoking and substance involvement screening test (ASSIST): manual for use in primary care.

[20] D’Zurilla TJ, Nezu AM (2010). Problem-solving therapy. Handb Cogn Ther..

[21] D’zurilla TJ, Goldfried MR (1971). Problem solving and behavior modification. J Abnorm Psychol..

[22] Bureau for economic research, Stellenosch University. Trends. 2012. www.ber.ac.za/data/2007.aspx. Accessed Sept 2013.

[23] Karnon J, Brown J (1998). Selecting a decision model for economic evaluation: a case study and review. Health Care Manag Sci..

[24] Briggs A (1995). Handling uncertainty in the results of economic evaluation. OHE Brief..

[25] Thokala P, Ochalek J, Leech AA, Tong T (2018). Cost-effectiveness thresholds: the past, the present and the future. Pharmacoeconomics..

[26] Buttorff C, Hock RS, Weiss HA, Naik S, Araya R, Kirkwood BR (2012). Economic evaluation of a task-shifting intervention for common mental disorders in India. Bull World Health Organ.

[27] Kakuma R, Minas H, van Ginneken N, Dal Poz MR, Desiraju K, Morris JE (2011). Human resources for mental health care: current situation and strategies for action. Lancet.

[28] Petersen I, Lund C, Bhana A, Flisher AJ (2012). A task shifting approach to primary mental health care for adults in South Africa: human resource requirements and costs for rural settings. Health Policy Plan..

[29] Barrett B, Byford S, Crawford MJ, Patton R, Drummond C, Henry JA (2006). Cost-effectiveness of screening and referral to an alcohol health worker in alcohol misusing patients attending an accident and emergency department: a decision-making approach. Drug Alcohol Depend.

[30] Kunz FM, French MT, Bazargan-Hejazi S (2004). Cost-effectiveness analysis of a brief intervention delivered to problem drinkers presenting at an inner-city hospital emergency department. J Stud Alcohol.

[31] Neighbors CJ, Barnett NP, Rohsenow DJ, Colby SM, Monti PM (2010). Cost-effectiveness of a motivational lntervention for alcohol-involved youth in a hospital emergency department. J Stud Alcohol Drugs..

[32] Birch S, Gafni A (2006). Information created to evade reality (ICER). Pharmacoeconomics..

[33] Cowell AJ, Brown JM, Mills MJ, Bender RH, Wedehase BJ (2012). Cost-effectiveness analysis of motivational interviewing with feedback to reduce drinking among a sample of college students. J Stud Alcohol Drugs..

[34] Cowell AJ, Bray JW, Mills MJ, Hinde JM (2010). Conducting economic evaluations of screening and brief intervention for hazardous drinking: methods and evidence to date for informing policy. Drug Alcohol Rev..

[35] Weinstein MC, Torrance G, McGuire A (2009). QALYs: the basics. Value Heal..

[36] Matzopoulos RG, Truen S, Bowman B, Corrigall J (2014). The cost of harmful alcohol use in South Africa. South Afr Med J (SAMJ).

